# Supplementary material for: Outcome of COVID-19 in hospitalised immunocompromised patients: An analysis of the WHO ISARIC CCP-UK prospective cohort study
Source: PLoS Med. 2023 Jan 31;20(1):e1004086. doi: 10.1371/journal.pmed.1004086 (PMC9928075; doi:10.1371/journal.pmed.1004086)
Supplement: S1 Table — (DOCX) [file pmed.1004086.s002.docx]

**S1 Table. Comorbidities in immunocompetent versus immunocompromised patients stratified by pandemic wave.** Data are numbers (%) except for age where median (IQR) is shown. Wave 1 was 17^th^ January 2020 to 31^st^ August 2020, wave 2 was from 1^st^ September 2020 to 31^st^ March 2021, wave 3 was from 1^st^ April 2021 to 12^th^ December 2021 and wave 4 was from 13^th^ December 2021 until the end of recruitment for the study on 28^th^ February 2022.

| label | levels | Immunocompetent - Wave 1 | Immunocompromised - Wave 1 | Immunocompetent - Wave 2 | Immunocompromised - Wave 2 | Immunocompetent - Wave 3 | Immunocompromised - Wave 3 | Immunocompetent – Wave 4 | Immunocompromised – Wave 4 |
| --- | --- | --- | --- | --- | --- | --- | --- | --- | --- |
| Age on admission (years) | Median (IQR) | 75.3 (59.7 to 84.9) | 74.1 (62.0 to 82.5) | 70.2 (55.6 to 81.9) | 71.3 (59.0 to 80.1) | 58.7 (42.2 to 75.2) | 66.9 (53.9 to 76.6) | 66.8 (45.5 to 81.5) | 69.2 (56.2 to 78.6) |
|  | <50 | 4716 (13.3) | 641 (9.3) | 10867 (16.8) | 1209 (12.3) | 9867 (36.4) | 806 (18.6) | 2174 (29.4) | 145 (15.7) |
|  | 50-69 | 9402 (26.5) | 2010 (29.3) | 21188 (32.8) | 3389 (34.4) | 8129 (30.0) | 1658 (38.3) | 1859 (25.2) | 330 (35.8) |
|  | 70-79 | 7472 (21.0) | 1977 (28.8) | 13751 (21.3) | 2764 (28.1) | 4381 (16.2) | 1110 (25.7) | 1298 (17.6) | 262 (28.4) |
|  | 80+ | 13931 (39.2) | 2230 (32.5) | 18755 (29.1) | 2485 (25.2) | 4738 (17.5) | 751 (17.4) | 2058 (27.9) | 185 (20.1) |
| Sex at Birth | Male | 19501 (54.9) | 3684 (53.7) | 36124 (56.0) | 5196 (52.8) | 15137 (55.8) | 2234 (51.7) | 3667 (49.6) | 450 (48.8) |
|  | Female | 15976 (45.0) | 3163 (46.1) | 28371 (43.9) | 4636 (47.1) | 11948 (44.1) | 2083 (48.2) | 3706 (50.2) | 471 (51.1) |
|  | Not specified | 41 (0.1) | 8 (0.1) | 60 (0.1) | 15 (0.2) | 22 (0.1) | 7 (0.2) | 13 (0.2) | 1 (0.1) |
| Ethnicity | White | 25322 (82.0) | 5207 (85.0) | 46492 (82.5) | 7399 (85.0) | 18501 (79.8) | 3262 (85.0) | 4945 (79.7) | 710 (86.8) |
|  | South Asian | 1856 (6.0) | 300 (4.9) | 4094 (7.3) | 490 (5.6) | 1610 (6.9) | 201 (5.2) | 418 (6.7) | 32 (3.9) |
|  | Black | 1159 (3.8) | 220 (3.6) | 1542 (2.7) | 229 (2.6) | 999 (4.3) | 127 (3.3) | 298 (4.8) | 27 (3.3) |
|  | East Asian | 243 (0.8) | 30 (0.5) | 307 (0.5) | 38 (0.4) | 158 (0.7) | 14 (0.4) | 22 (0.4) | 1 (0.1) |
|  | Other | 2313 (7.5) | 368 (6.0) | 3920 (7.0) | 547 (6.3) | 1924 (8.3) | 233 (6.1) | 522 (8.4) | 48 (5.9) |
| Number of comorbidities | 0 | 4378 (12.3) | 285 (4.2) | 10230 (15.8) | 489 (5.0) | 8238 (30.4) | 335 (7.7) | 1762 (23.8) | 44 (4.8) |
|  | 1 | 6868 (19.3) | 979 (14.3) | 13649 (21.1) | 1599 (16.2) | 5809 (21.4) | 700 (16.2) | 1458 (19.7) | 157 (17.0) |
|  | 2+ | 24279 (68.3) | 5594 (81.6) | 40688 (63.0) | 7760 (78.8) | 13070 (48.2) | 3291 (76.1) | 4169 (56.4) | 721 (78.2) |
| Chronic Cardiac Disease | No | 22615 (66.7) | 4261 (64.8) | 43530 (71.4) | 6571 (69.2) | 15241 (75.5) | 2924 (72.8) | 4004 (68.1) | 595 (68.0) |
|  | Yes | 11298 (33.3) | 2315 (35.2) | 17470 (28.6) | 2918 (30.8) | 4955 (24.5) | 1094 (27.2) | 1874 (31.9) | 280 (32.0) |
| Hypertension | No | 17156 (50.4) | 3495 (53.1) | 32532 (52.9) | 5060 (53.2) | 11717 (57.5) | 2176 (54.1) | 3290 (55.7) | 500 (57.3) |
|  | Yes | 16889 (49.6) | 3083 (46.9) | 28933 (47.1) | 4456 (46.8) | 8674 (42.5) | 1846 (45.9) | 2621 (44.3) | 373 (42.7) |
| Chronic Pulmonary Disease | No | 28521 (84.6) | 4602 (69.9) | 52034 (85.3) | 6752 (70.8) | 17117 (84.5) | 2920 (72.3) | 4736 (80.2) | 581 (65.6) |
|  | Yes | 5189 (15.4) | 1983 (30.1) | 8999 (14.7) | 2784 (29.2) | 3138 (15.5) | 1118 (27.7) | 1169 (19.8) | 305 (34.4) |
| Chronic Renal Disease | No | 27710 (82.3) | 5149 (78.8) | 51719 (85.0) | 7677 (81.1) | 17395 (86.2) | 3132 (78.0) | 4873 (82.7) | 670 (76.4) |
|  | Yes | 5961 (17.7) | 1386 (21.2) | 9136 (15.0) | 1791 (18.9) | 2784 (13.8) | 882 (22.0) | 1020 (17.3) | 207 (23.6) |
| Asthma | No | 29546 (87.7) | 5269 (80.7) | 52021 (85.4) | 7369 (77.5) | 16569 (81.7) | 3138 (77.6) | 4952 (84.0) | 700 (79.5) |
|  | Yes | 4150 (12.3) | 1257 (19.3) | 8922 (14.6) | 2140 (22.5) | 3705 (18.3) | 905 (22.4) | 943 (16.0) | 181 (20.5) |
| Liver Disease | No | 32281 (96.7) | 6204 (95.8) | 58724 (96.9) | 8987 (95.5) | 19463 (96.8) | 3833 (95.8) | 5612 (96.6) | 825 (95.3) |
|  | Yes | 1112 (3.3) | 274 (4.2) | 1856 (3.1) | 422 (4.5) | 635 (3.2) | 168 (4.2) | 197 (3.4) | 41 (4.7) |
| Chronic Neurological Disorder | No | 28925 (86.2) | 5729 (88.1) | 54021 (89.0) | 8478 (89.9) | 18345 (90.9) | 3691 (92.0) | 5049 (86.4) | 779 (89.1) |
|  | Yes | 4640 (13.8) | 776 (11.9) | 6678 (11.0) | 953 (10.1) | 1844 (9.1) | 321 (8.0) | 795 (13.6) | 95 (10.9) |
| Malignant Neoplasm | No | 31019 (92.8) | 4857 (74.6) | 56420 (93.0) | 7206 (76.2) | 18895 (93.8) | 3048 (76.2) | 5269 (90.5) | 628 (71.4) |
|  | Yes | 2423 (7.2) | 1656 (25.4) | 4256 (7.0) | 2252 (23.8) | 1250 (6.2) | 953 (23.8) | 555 (9.5) | 252 (28.6) |
| Chronic Haemotologic Disease | No | 32397 (96.9) | 5796 (89.4) | 58907 (97.1) | 8596 (91.2) | 19535 (96.9) | 3600 (89.6) | 5592 (95.9) | 766 (87.8) |
|  | Yes | 1039 (3.1) | 690 (10.6) | 1761 (2.9) | 829 (8.8) | 623 (3.1) | 416 (10.4) | 242 (4.1) | 106 (12.2) |
| Obesity | No | 26042 (87.3) | 5016 (87.5) | 44460 (82.9) | 6693 (81.8) | 14327 (79.0) | 2886 (82.0) | 4728 (87.3) | 705 (87.0) |
|  | Yes | 3793 (12.7) | 717 (12.5) | 9167 (17.1) | 1489 (18.2) | 3815 (21.0) | 634 (18.0) | 687 (12.7) | 105 (13.0) |
| Diabetes | No | 23631 (69.0) | 4669 (70.3) | 43534 (70.7) | 6874 (71.7) | 14424 (70.6) | 2915 (71.8) | 4255 (71.9) | 677 (76.2) |
|  | Yes | 10602 (31.0) | 1970 (29.7) | 18061 (29.3) | 2716 (28.3) | 5995 (29.4) | 1145 (28.2) | 1664 (28.1) | 211 (23.8) |
| Rheumatologic Disorder | No | 29815 (89.5) | 5105 (78.8) | 53567 (88.7) | 7351 (77.9) | 18101 (90.1) | 3116 (77.5) | 5279 (90.3) | 701 (79.8) |
|  | Yes | 3487 (10.5) | 1374 (21.2) | 6858 (11.3) | 2084 (22.1) | 1992 (9.9) | 905 (22.5) | 564 (9.7) | 177 (20.2) |
| Dementia | No | 26981 (80.4) | 5772 (88.7) | 53735 (89.0) | 8762 (93.3) | 18717 (93.1) | 3834 (95.9) | 5130 (87.9) | 837 (94.9) |
|  | Yes | 6588 (19.6) | 735 (11.3) | 6651 (11.0) | 629 (6.7) | 1380 (6.9) | 165 (4.1) | 709 (12.1) | 45 (5.1) |
| Malnutrition | No | 30161 (97.0) | 5841 (97.0) | 55152 (97.9) | 8391 (97.1) | 18759 (98.8) | 3696 (98.7) | 5459 (98.3) | 821 (98.0) |
|  | Yes | 929 (3.0) | 181 (3.0) | 1164 (2.1) | 249 (2.9) | 227 (1.2) | 47 (1.3) | 97 (1.7) | 17 (2.0) |
| Smoking | No | 9736 (52.1) | 1741 (44.1) | 18467 (52.5) | 2578 (45.1) | 5785 (47.8) | 1082 (44.5) | 1390 (43.2) | 186 (35.6) |
|  | Yes | 8969 (47.9) | 2206 (55.9) | 16682 (47.5) | 3132 (54.9) | 6319 (52.2) | 1352 (55.5) | 1828 (56.8) | 336 (64.4) |
| Vaccination Dose on Admission | Unvaccinated | 34396 (100.0) | 6688 (100.0) | 59598 (94.9) | 9143 (94.4) | 13488 (49.7) | 1058 (24.5) | 6882 (93.1) | 848 (92.0) |
|  | First Dose | 0 (0.0) | 0 (0.0) | 3116 (5.0) | 532 (5.5) | 1948 (7.2) | 253 (5.8) | 48 (0.6) | 4 (0.4) |
|  | Second Dose | 0 (0.0) | 0 (0.0) | 55 (0.1) | 11 (0.1) | 11251 (41.5) | 2762 (63.8) | 295 (4.0) | 31 (3.4) |
|  | Third Dose | 0 (0.0) | 0 (0.0) | 0 (0.0) | 0 (0.0) | 428 (1.6) | 251 (5.8) | 164 (2.2) | 39 (4.2) |
|  | Fourth Dose | 0 (0.0) | 0 (0.0) | 0 (0.0) | 0 (0.0) | 2 (0.0) | 2 (0.0) | 0 (0.0) | 0 (0.0) |
