# Supplementary material for: Outcome of COVID-19 in hospitalised immunocompromised patients: An analysis of the WHO ISARIC CCP-UK prospective cohort study
Source: PLoS Med. 2023 Jan 31;20(1):e1004086. doi: 10.1371/journal.pmed.1004086 (PMC9928075; doi:10.1371/journal.pmed.1004086)
Supplement: S2 Table — (DOCX) [file pmed.1004086.s003.docx]

**S2 Table. Disease severity by immune status and pandemic wave.** The 4C mortality score is calculated on admission to hospital from age, sex, number of comorbidities, respiratory rate, oxygen saturation, Glasgow coma scale, urea and CRP. The score ranges from 0 (low risk) to 21 (very high risk). Score 0-3 = low risk (1.2% mortality), 4-8 = intermediate risk (9.9% mortality), 9-14 = high risk (31.4% mortality) and 15-21 = very high risk (61.5% mortality).

| label | levels | Immunocompetent - Wave 1 | Immunocompromised - Wave 1 | Immunocompetent - Wave 2 | Immunocompromised - Wave 2 | Immunocompetent - Wave 3 | Immunocompromised - Wave 3 | Immunocompetent – Wave 4 | Immunocompromised – Wave 4 |
| --- | --- | --- | --- | --- | --- | --- | --- | --- | --- |
| ISARIC4C Mortality Score | Median (IQR) | 11.0 (8.0 to 13.0) | 11.0 (9.0 to 13.0) | 10.0 (7.0 to 13.0) | 11.0 (8.0 to 13.0) | 8.0 (4.0 to 11.0) | 10.0 (7.0 to 12.0) | 9.0 (4.0 to 12.0) | 10.0 (7.0 to 12.0) |
| Respiratory Rate (breaths per min) | <20 | 12159 (35.1) | 2259 (33.6) | 20832 (33.1) | 3060 (31.6) | 8563 (32.5) | 1291 (30.3) | 2453 (42.6) | 283 (33.0) |
|  | 20-30 | 16963 (48.9) | 3382 (50.4) | 33071 (52.5) | 5173 (53.5) | 14210 (54.0) | 2406 (56.5) | 2726 (47.4) | 463 (54.0) |
|  | >=30 | 5558 (16.0) | 1074 (16.0) | 9102 (14.4) | 1439 (14.9) | 3545 (13.5) | 564 (13.2) | 578 (10.0) | 112 (13.1) |
| Number of comorbidities | 0 | 4378 (12.3) | 285 (4.2) | 10230 (15.8) | 489 (5.0) | 8238 (30.4) | 335 (7.7) | 1762 (23.8) | 44 (4.8) |
|  | 1 | 6868 (19.3) | 979 (14.3) | 13649 (21.1) | 1599 (16.2) | 5809 (21.4) | 700 (16.2) | 1458 (19.7) | 157 (17.0) |
|  | 2+ | 24279 (68.3) | 5594 (81.6) | 40688 (63.0) | 7760 (78.8) | 13070 (48.2) | 3291 (76.1) | 4169 (56.4) | 721 (78.2) |
| Oxygen Saturation (%) | >=92 | 27589 (79.2) | 5254 (77.7) | 49645 (78.6) | 7421 (76.6) | 21201 (80.0) | 3323 (77.9) | 4912 (84.9) | 671 (77.7) |
|  | <92 | 7244 (20.8) | 1507 (22.3) | 13495 (21.4) | 2262 (23.4) | 5288 (20.0) | 942 (22.1) | 875 (15.1) | 193 (22.3) |
| Glasgow Coma Score | 15 | 27882 (84.5) | 5664 (88.2) | 55467 (90.9) | 8585 (91.8) | 24134 (93.9) | 3872 (94.0) | 5403 (91.0) | 771 (92.2) |
|  | <15 | 5128 (15.5) | 756 (11.8) | 5534 (9.1) | 767 (8.2) | 1562 (6.1) | 249 (6.0) | 533 (9.0) | 65 (7.8) |
| Blood Urea (mmol/L) | <7 | 13721 (48.4) | 2633 (47.2) | 28504 (54.9) | 4109 (50.9) | 14805 (66.2) | 1877 (51.3) | 3024 (61.3) | 378 (51.5) |
|  | 7-14 | 9659 (34.0) | 2027 (36.3) | 17073 (32.9) | 2927 (36.3) | 5636 (25.2) | 1291 (35.3) | 1355 (27.5) | 258 (35.1) |
|  | >14 | 4998 (17.6) | 919 (16.5) | 6320 (12.2) | 1032 (12.8) | 1927 (8.6) | 490 (13.4) | 557 (11.3) | 98 (13.4) |
| C-Reactive Protein (mg/L) | <50 | 10342 (35.9) | 1932 (33.9) | 18415 (35.5) | 2658 (32.8) | 8349 (36.8) | 1261 (33.9) | 2552 (52.8) | 307 (42.1) |
|  | 50-99 | 6632 (23.0) | 1400 (24.6) | 13312 (25.7) | 2160 (26.7) | 5556 (24.5) | 1005 (27.0) | 892 (18.5) | 167 (22.9) |
|  | >=100 | 11824 (41.1) | 2363 (41.5) | 20110 (38.8) | 3277 (40.5) | 8757 (38.6) | 1455 (39.1) | 1385 (28.7) | 256 (35.1) |
