# Supplementary material for: Outcome of COVID-19 in hospitalised immunocompromised patients: An analysis of the WHO ISARIC CCP-UK prospective cohort study
Source: PLoS Med. 2023 Jan 31;20(1):e1004086. doi: 10.1371/journal.pmed.1004086 (PMC9928075; doi:10.1371/journal.pmed.1004086)
Supplement: S3 Table — (DOCX) [file pmed.1004086.s004.docx]

**S3 Table. Number (%) of patients receiving steroids and tocilizumab by immune status and pandemic wave.** Data are numbers of patients (%).

| label | levels | Immunocompetent - Wave 1 | Immunocompromised - Wave 1 | Immunocompetent - Wave 2 | Immunocompromised - Wave 2 | Immunocompetent - Wave 3 | Immunocompromised - Wave 3 | Immunocompetent – Wave 4 | Immunocompromised – Wave 4 |
| --- | --- | --- | --- | --- | --- | --- | --- | --- | --- |
| Steroids | No | 20192 (83.3) | 2405 (48.9) | 7721 (16.3) | 824 (10.9) | 2223 (11.7) | 277 (8.5) | 1246 (36.3) | 101 (18.7) |
|  | Yes | 4035 (16.7) | 2513 (51.1) | 39747 (83.7) | 6725 (89.1) | 16776 (88.3) | 2981 (91.5) | 2188 (63.7) | 439 (81.3) |
| Tocilizumab | No | 12340 (99.5) | 2482 (99.6) | 21610 (93.7) | 3529 (95.5) | 7812 (80.4) | 1422 (85.0) | 1107 (86.7) | 206 (88.8) |
|  | Yes | 59 (0.5) | 11 (0.4) | 1456 (6.3) | 167 (4.5) | 1900 (19.6) | 251 (15.0) | 170 (13.3) | 26 (11.2) |
