# Supplementary material for: Outcome of COVID-19 in hospitalised immunocompromised patients: An analysis of the WHO ISARIC CCP-UK prospective cohort study
Source: PLoS Med. 2023 Jan 31;20(1):e1004086. doi: 10.1371/journal.pmed.1004086 (PMC9928075; doi:10.1371/journal.pmed.1004086)
Supplement: S4 Table — (DOCX) [file pmed.1004086.s005.docx]

**S4 Table. Outcomes stratified by immune status and pandemic wave.** The number of immunocompetent and immunocompromised patients requiring oxygen, critical care admission, non-invasive ventilation, invasive ventilation and death across the 4 pandemic waves of the study. In brackets is the percentage for each outcome in each group of patients, per pandemic wave. Wave 1 was 17^th^ January 2020 to 31^st^ August 2020, wave 2 was from 1^st^ September 2020 to 31^st^ March 2021, wave 3 was from 1^st^ April 2021 to 12^th^ December 2021 and wave 4 was from 13^th^ December 2021 until the end of recruitment for the study on 28^th^ February 2022.

| label | levels | Immunocompetent - Wave 1 | Immunocompromised - Wave 1 | Immunocompetent - Wave 2 | Immunocompromised - Wave 2 | Immunocompetent - Wave 3 | Immunocompromised - Wave 3 | Immunocompetent – Wave 4 | Immunocompromised – Wave 4 |
| --- | --- | --- | --- | --- | --- | --- | --- | --- | --- |
| Oxygen | No | 10974 (31.2) | 1910 (28.0) | 16398 (25.7) | 2227 (22.8) | 7745 (29.0) | 1029 (24.0) | 3659 (51.6) | 361 (40.1) |
|  | Yes | 24227 (68.8) | 4918 (72.0) | 47468 (74.3) | 7549 (77.2) | 18999 (71.0) | 3258 (76.0) | 3434 (48.4) | 540 (59.9) |
| Critical Care Admission | No | 30785 (86.9) | 5952 (86.9) | 52873 (82.5) | 8101 (82.7) | 22469 (83.9) | 3514 (82.0) | 6611 (92.4) | 823 (90.8) |
|  | Yes | 4637 (13.1) | 896 (13.1) | 11223 (17.5) | 1693 (17.3) | 4306 (16.1) | 773 (18.0) | 544 (7.6) | 83 (9.2) |
| Non-Invasive Ventilation | No | 29970 (85.4) | 5660 (83.2) | 50363 (79.4) | 7449 (76.8) | 20925 (78.9) | 3181 (74.7) | 6362 (90.1) | 762 (84.9) |
|  | Yes | 5106 (14.6) | 1145 (16.8) | 13035 (20.6) | 2252 (23.2) | 5608 (21.1) | 1078 (25.3) | 696 (9.9) | 136 (15.1) |
| Invasive Ventilation | No | 32524 (92.4) | 6384 (93.6) | 59058 (93.0) | 8973 (92.2) | 25074 (94.7) | 3948 (92.8) | 6875 (97.6) | 866 (96.7) |
|  | Yes | 2664 (7.6) | 435 (6.4) | 4416 (7.0) | 754 (7.8) | 1415 (5.3) | 308 (7.2) | 170 (2.4) | 30 (3.3) |
| Death | No | 24766 (70.2) | 4379 (64.3) | 48439 (77.4) | 6551 (68.6) | 23276 (87.9) | 3317 (78.3) | 5726 (88.7) | 662 (81.2) |
|  | Yes | 10495 (29.8) | 2430 (35.7) | 14177 (22.6) | 2998 (31.4) | 3210 (12.1) | 918 (21.7) | 726 (11.3) | 153 (18.8) |
