# Supplementary material for: Outcome of COVID-19 in hospitalised immunocompromised patients: An analysis of the WHO ISARIC CCP-UK prospective cohort study
Source: PLoS Med. 2023 Jan 31;20(1):e1004086. doi: 10.1371/journal.pmed.1004086 (PMC9928075; doi:10.1371/journal.pmed.1004086)

**S1 Figure. Number of admissions of immunocompetent and immunocompromised patients over time.** The number of patients admitted over time is indicated for immunocompetent (pink) and immunocompromised (turquoise) patients. The dashed lines indicate 95% confidence interval. The dotted vertical line represents the date of introduction of the vaccine program in the UK.


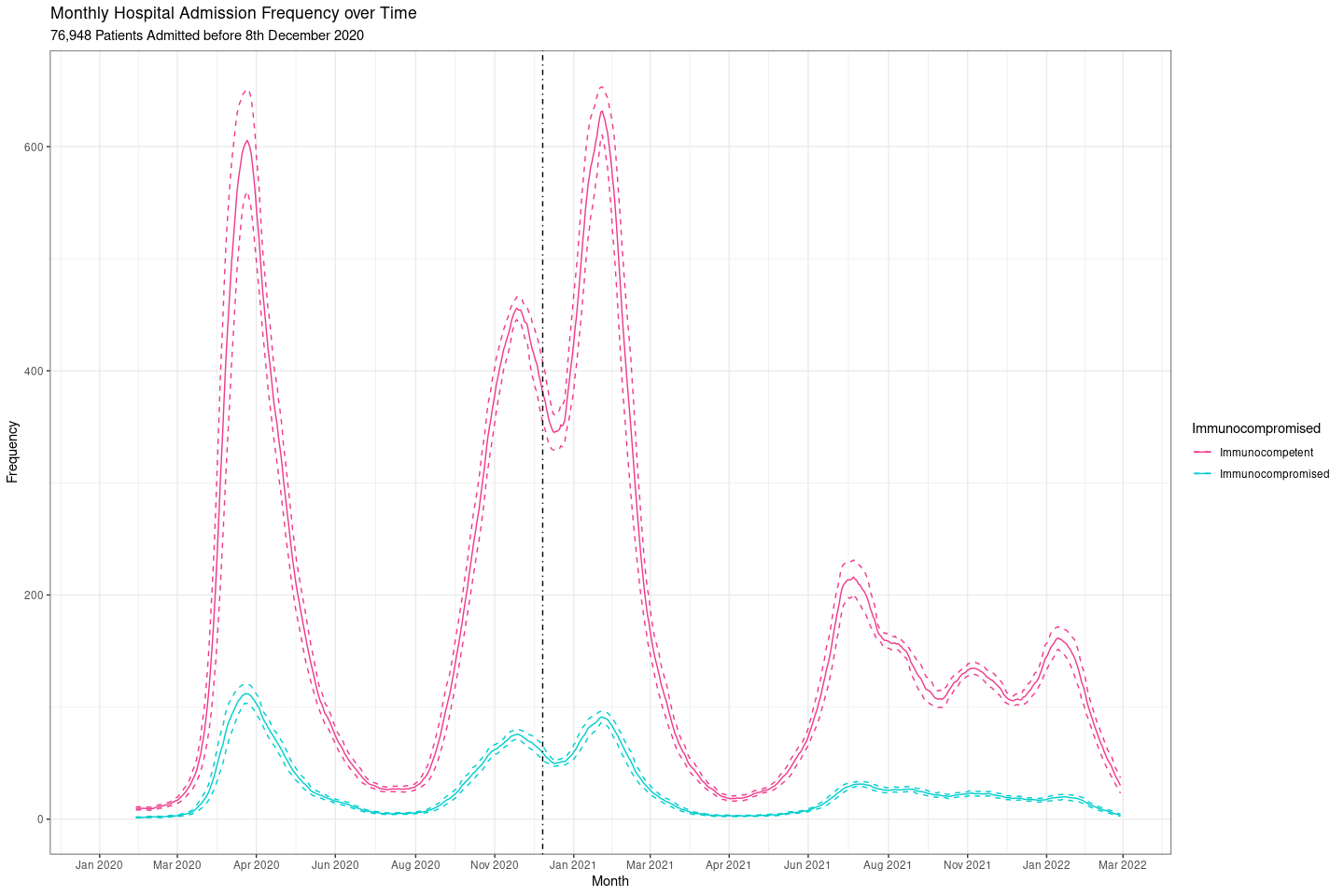

Supplement: S1 Fig — (DOCX) [file pmed.1004086.s006.docx]
