# Supplementary material for: Outcome of COVID-19 in hospitalised immunocompromised patients: An analysis of the WHO ISARIC CCP-UK prospective cohort study
Source: PLoS Med. 2023 Jan 31;20(1):e1004086. doi: 10.1371/journal.pmed.1004086 (PMC9928075; doi:10.1371/journal.pmed.1004086)

**S2 Figure. Admissions stratified by the number of vaccine doses received.** Rolling monthly average hospital admissions by number of vaccine doses received for immunocompetent (left) and immunocompromised (right) patients.


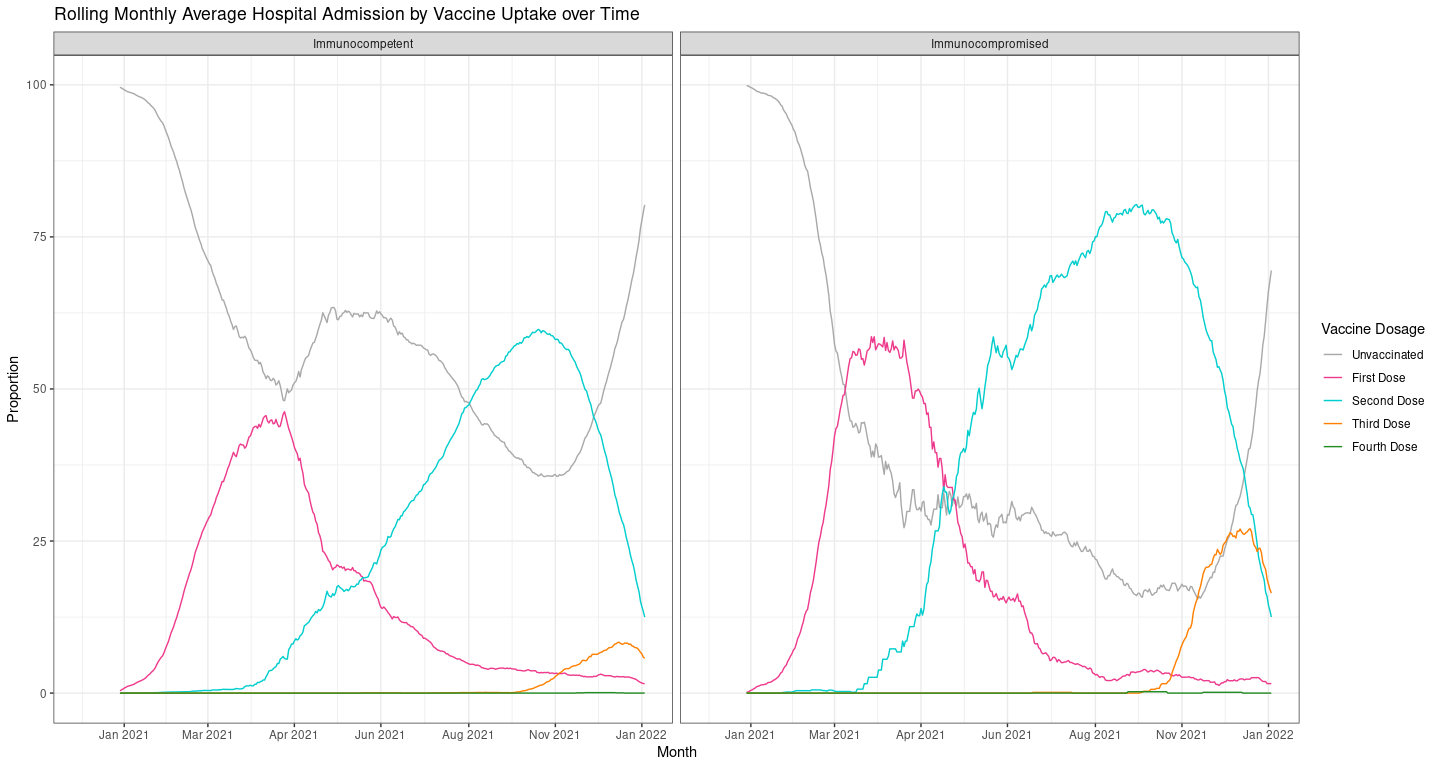

Supplement: S2 Fig — (DOCX) [file pmed.1004086.s007.docx]
