# Supplementary material for: Outcome of COVID-19 in hospitalised immunocompromised patients: An analysis of the WHO ISARIC CCP-UK prospective cohort study
Source: PLoS Med. 2023 Jan 31;20(1):e1004086. doi: 10.1371/journal.pmed.1004086 (PMC9928075; doi:10.1371/journal.pmed.1004086)

**S4 Figure. Tocilizumab usage by immune status in the first 4 pandemic waves in the UK.** The proportion of immunocompetent and immunocompromised patients with available data in each wave who received tocilizumab in hospital. The number of patients with available data are: wave 1, immunocompetent 12399, immunocompromised 2493; wave 2, immunocompetent 23066, immunocompromised 3696; wave 3, immunocompetent 9712, immunocompromised 1673; wave 4, immunocompetent 1277, immunocompromised 232.


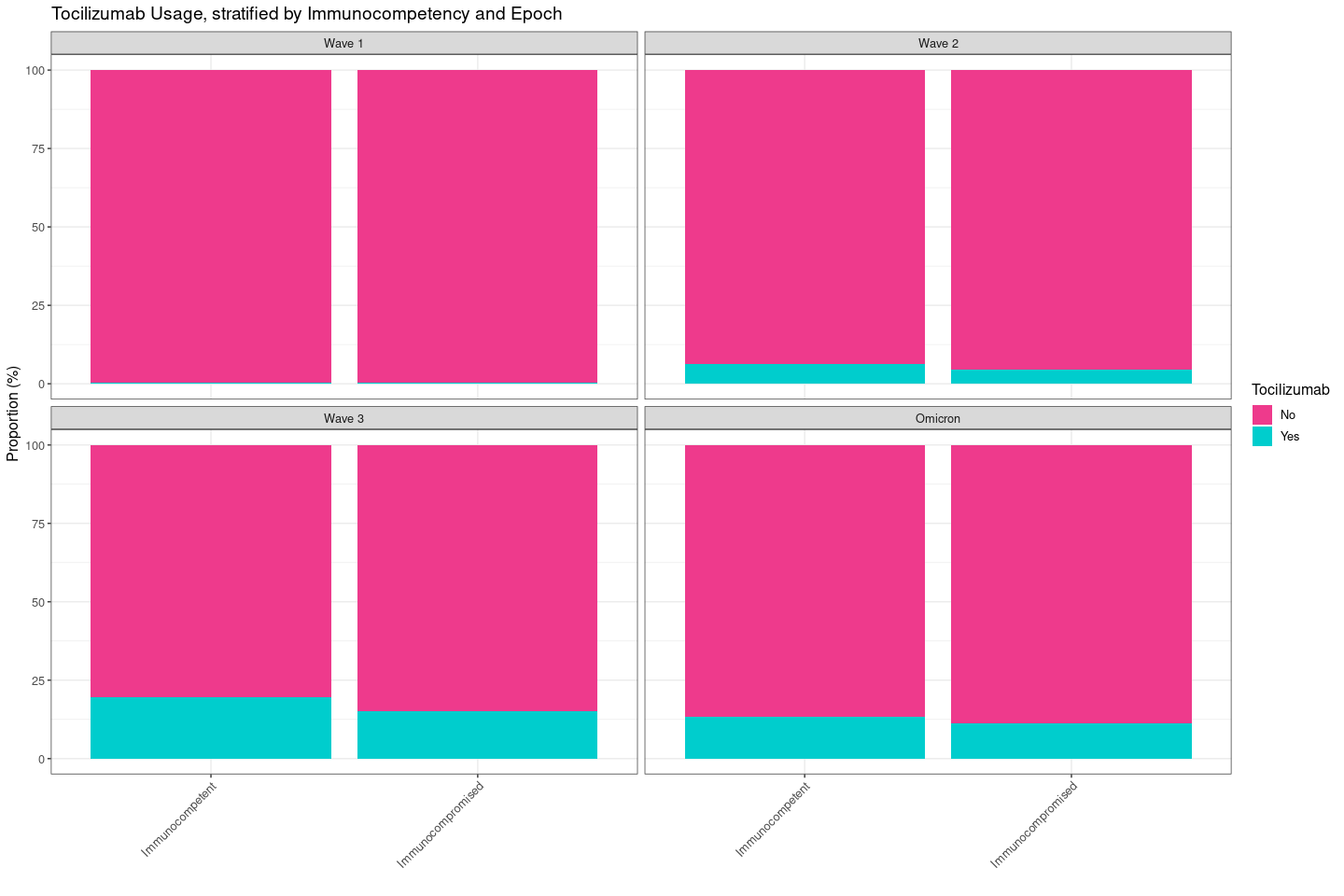

Supplement: S4 Fig — (DOCX) [file pmed.1004086.s009.docx]
