# Supplementary material for: Outcome of COVID-19 in hospitalised immunocompromised patients: An analysis of the WHO ISARIC CCP-UK prospective cohort study
Source: PLoS Med. 2023 Jan 31;20(1):e1004086. doi: 10.1371/journal.pmed.1004086 (PMC9928075; doi:10.1371/journal.pmed.1004086)

**S5 Figure.** **Outcome of hospitalised immunocompromised patients, compared with immunocompetent patients – univariable analysis.** Odds ratios (OR) for in-hospital death from univariable logistic regression in each of the first four pandemic waves in the UK. Bars represent the 95% confidence interval.


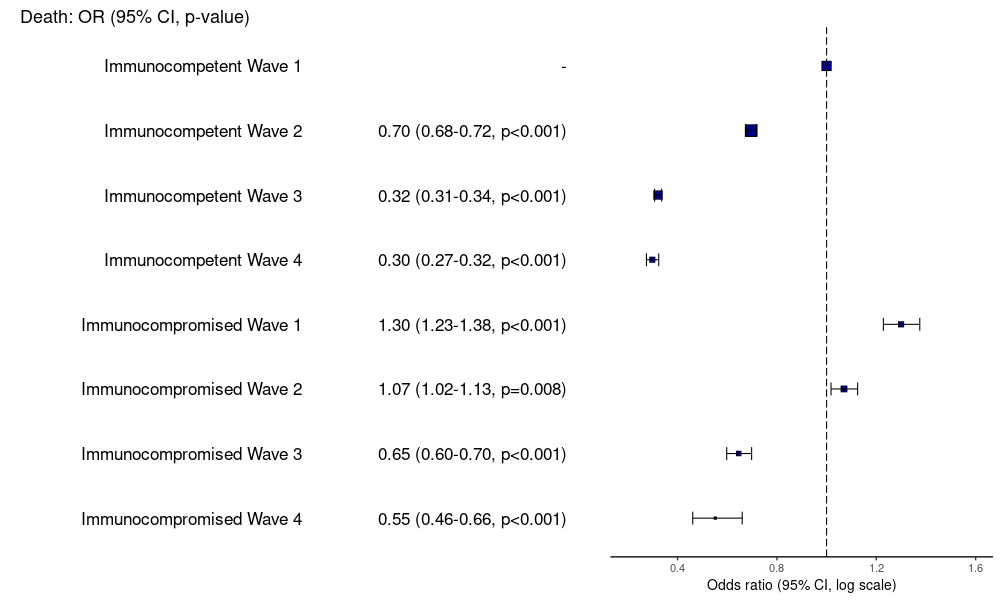

Supplement: S5 Fig — (DOCX) [file pmed.1004086.s010.docx]
