# Supplementary material for: Outcome of COVID-19 in hospitalised immunocompromised patients: An analysis of the WHO ISARIC CCP-UK prospective cohort study
Source: PLoS Med. 2023 Jan 31;20(1):e1004086. doi: 10.1371/journal.pmed.1004086 (PMC9928075; doi:10.1371/journal.pmed.1004086)

**S6 Figure. Sensitivity analysis for the primary and secondary outcomes using imputation of missing data.** ORs from multivariable logistic regression, adjusted for age, sex, ethnicity, socioeconomic deprivation, chronic cardiac, pulmonary and renal disease, and vaccination status.


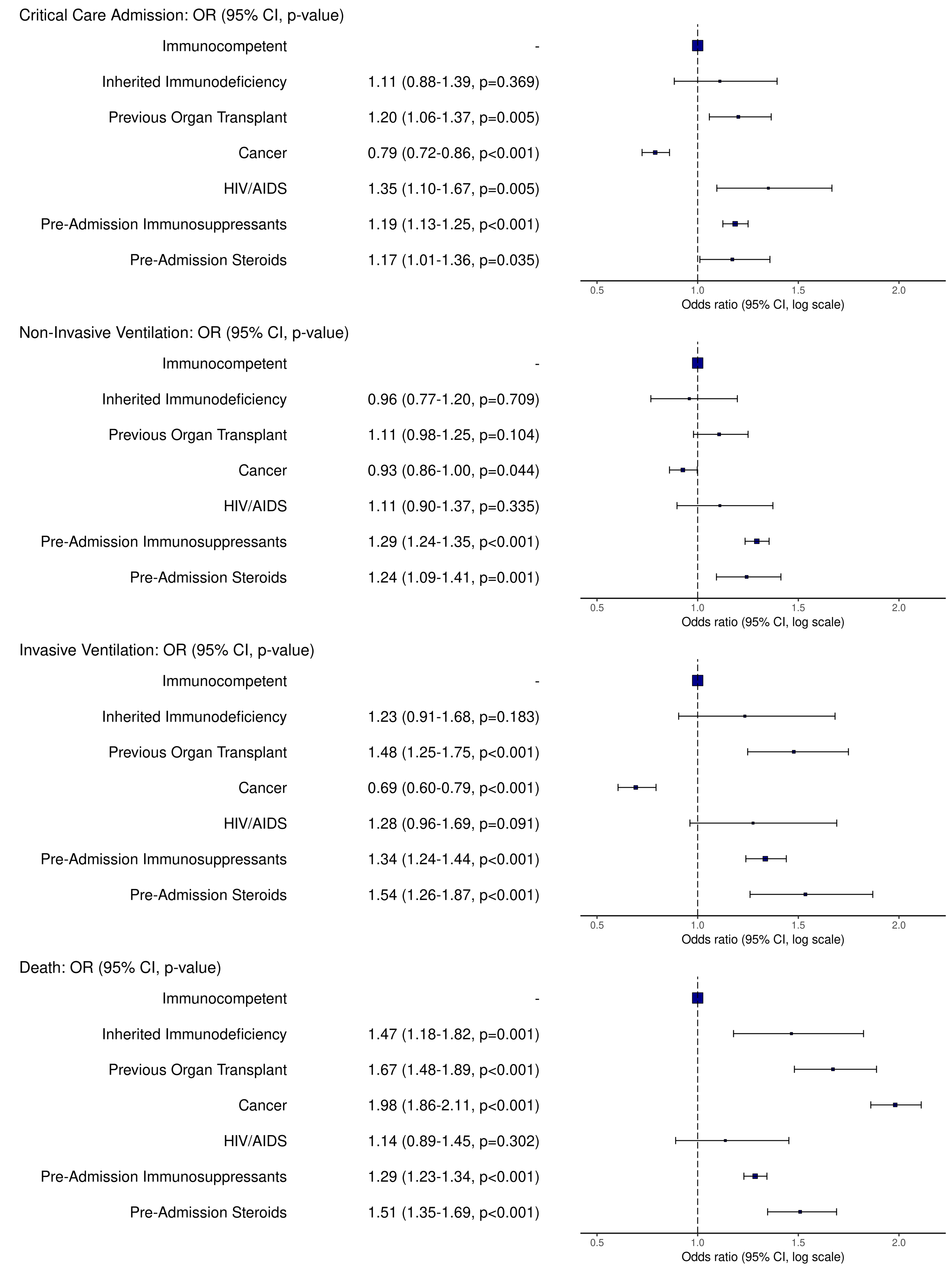

Supplement: S6 Fig — (DOCX) [file pmed.1004086.s011.docx]
