# Supplementary material for: Outcome of COVID-19 in hospitalised immunocompromised patients: An analysis of the WHO ISARIC CCP-UK prospective cohort study
Source: PLoS Med. 2023 Jan 31;20(1):e1004086. doi: 10.1371/journal.pmed.1004086 (PMC9928075; doi:10.1371/journal.pmed.1004086)
Supplement: Attachment — Submitted filename: turtle.pdf [file pmed.1004086.s013.pdf]

This paper compares the outcomes for people admitted to hospitals with COVID–19 in the UK and the association with immunocompromise.

I was asked for a statistical report and I interpret that to include all aspects of the design and conduct of the study.

Points of detail

Page 5 The usual definition of adulthood in the UK is ≥ 18. Is this a typo?

Page 6 I think we might need more explanation of what hospital level deprivation is. Is this a single figure which tries to summarise the entire hospital catchment area? Is it the deprivation index for the middle layer super output area within which the hospital is located? Perhaps more importantly is there any evidence that such a number is related to important features of health outcomes?

Page 7 The frequentist and Bayesian analyses seem to have used a different set of covariates. Vaccination is missing in the Bayesian one. A sentence explaining why might be useful.

Page 7 It would be good to give citations for some of the packages. It is the main way authors get credit. (Disclosure: I am a CRAN package author but have no connection with any of these.)

Page 19 Typo in caption 'as both the, incorporating'?

Figure 1 The labelling here seems to be in the reverse order to the labelling of the sub–figures. I would have to say I did not find the Euler diagram either easy to read or very enlightening. It is hard to see how much overlap there is assuming that is what the authors intend me to see. The authors might want to consider other packages like `eulerr` which has some extensive vignettes or, for a different and perhaps more enlightening approach, `UpSetR`.

Figure 2 If the waves differ so much is it informative to present results in sub–figure A collapsed over wave?

Supplementary Table S1 Why do so many people in wave 4 appear to be unvaccinated (90+%)? Was vaccination so effective by that time point that it prevented so many admissions?

Supplementary Figure S6 What do the dashed lines represent?

Points of more substance

Missingness

On page 7 the authors state that since their analysis is predominantly descriptive they are using not taking any steps to deal with missing data. In a sense any true empirical statement can be called descriptive, it describes the world as it is, but that does not mean we can ignore the problem of missing data by default. Clearly I do not find the authors' justification here very convincing.

The crucial information which we need to evaluate what is happeing is the extent to which the analysis sample is reduced. As far as I can see this is not presented anywhere, for instance in Figure S1 (which I would have thought important enough to be in the main article. Since the frequentist and Bayesian analysis appear to have used different predictor variables we need it for both analysis sets. Looking at Table 1 it does seem that some of the missingness may be related to immunocompromise and so there is a potential for bias.

At the least we need to know what variables predict missingness and if it does turn out not to be completely missing at random then imputation would seem indicated. Just to be clear I am not whingeing about the amount of missing. I believe most of the data was collected by non–professional researchers working under difficult circumstances and it is remarkable how little is missing.

Adjusting for comorbidities

I am not clear from Table 1 whether the tally for comorbidity includes the condition(s) which have led to immunocompromise. It is rather suspicious that nobody in that group is free of comobidity. One of the statements on page 8 seems to confirm that this is indeed a structural zero. Surely if it is genuinely *co*morbidity it should not include the immune problem itself? Either way I think more thought is needed here. Categorising so that 90+% fall into the 2+ category is too coarse for us to see what the issues are here but adjusting for a variable with very different distributions in the groups reminds me of Lord's paradox (Lord, 1967).

The secondary outcomes

If these outcomes are related to mortality, which there must be evidence that they are, then I find it odd that the odds ratios reported here are less than

for mortality itself. Is there some explanation for the differences? I am no expert on critical care so my surprise may be based purely on ignorance.

Summary

Points for clarification and some puzzles about the analysis.

Michael Dewey

References

F M Lord. A paradox in the interpretation of group comparisons. *Psychological Bulletin*, 68:304–305, 1967.
