# Supplementary material for: Outcome of COVID-19 in hospitalised immunocompromised patients: An analysis of the WHO ISARIC CCP-UK prospective cohort study
Source: PLoS Med. 2023 Jan 31;20(1):e1004086. doi: 10.1371/journal.pmed.1004086 (PMC9928075; doi:10.1371/journal.pmed.1004086)
Supplement: Attachment — Submitted filename: Response to reviewers PMEDICINE-D-22-02623R1.docx [file pmed.1004086.s014.docx]

**Response to comments from the reviewers**

Below, the comments from the reviewers and the academic editor are given in black. The authors’ responses to these points are given in blue.

**Reviewer #1:**

This work evaluates the impact of immunosuppression on the outcome of COVID-19 patients admitted to the hospital using the large WHO ISARIC CCP-UK cohort involving the four waves of the pandemic in Wales and England. This is a very relevant issue with important potential consequences in the design of preventive policies to protect this specific collective. Results from this study support that the presence of a basal status of immunosuppression is a risk factor for hospital mortality and to lesser extent to other outcomes indicating severity (receiving oxygen, critical care admission, non-invasive ventilation and invasive ventilation). Although the mortality decreased with the pandemic course, this decrease was less patent in those patients with immunosuppression. This reviewer has some comments/suggestions.

1. In the methods section, the authors state that "To examine in-hospital mortality, we initially performed multivariable logistic regression adjusted for age, sex, number of comorbidities, ethnicity, deprivation index and vaccination". Nonetheless, in the Fig 2b legend, the authors state that "ORs are adjusted for age, sex, ethnicity, deprivation, and comorbidities". Why vaccination is gone here?

Authors’ response: vaccination was indeed included as a co-variate in the logistic regression model, this is an omission which we have corrected. We are grateful to the reviewer for picking this up. The Figure 2 legend (which is now Figure 3 in the revised manuscript because of the change of Figure S1 to Figure 1).

2. I wonder if the association between immunosuppression and mortality is still present after adjustment by the pharmacological treatments received during hospitalization: steroids, tocilizumab, remdesivir...If this analysis is not feasible, I suggest acknowledging it as a limitation.

Authors’ response: We thank the reviewer for picking up this good point. These data are not complete enough unfortunately for this analysis to be conducted. We have added this limitation to the discussion. Of note, the relationship between immunocompromise and poor outcome is also present in the first wave, before these treatments were in widespread use.

3. Comorbidities are introduced in the model as "number of comorbidities", instead of as individual entities. Please justify it. If an aggregated score is used, is it possible to use the Charlson score?

Authors’ response: Co-morbidity count is used because previous work has shown that the number of comorbidities to be a good predictor of mortality, and it makes the model simpler. Co-morbidity count is the variable used in the ISARIC4C mortality score, and it performs better than individual co-morbidities (Knight et al BMJ 2020, ref 18). The ISARIC CCP-UK CRF did not collect sufficient detail to calculate the Charlson comorbidity index, eg cardiac disease is a single field. In addition, we have altered our analysis in Figure 3A where we now present ORs adjusted by the most important individual comorbidities, chronic cardiac, pulmonary, and renal disease. We have also changed the way we are counting the comorbidities (see table 1), by removing the immunocompromising condition from the comorbidity count. None of these changes have made any substantial difference to the conclusions of the study.

4. In the methods section, the authors state that during the first wave, highly suspected cases were also eligible for inclusion, because SARS-CoV-2 was an emergent pathogen at that time and laboratory confirmation was dependent on availability of testing. Are the results the same excluding these patients from the analysis, I mean, maintaining only those with a confirmed SARS-CoV-2 infection? A recent study has demonstrated that while COVID-19 disease was a large component of total aLRTD during the pandemic period, non- SARS-CoV-2 infection still caused the majority of respiratory infection hospitalisations (DOI:https://doi.org/10.1016/j.lanepe.2022.100473)

Authors’ response: We acknowledge this is a limitation which affects the first wave. However, the recruitment criteria were not simply pneumonia, they were clinical suspicion of COVID-19 at that point in the pandemic. Many UK clinicians felt that they could clinically distinguish COVID from other causes of pneumonia at least in obvious cases (the kind of cases that would have been recruited under these criteria). Moreover, although we may have overestimated the number of these patients that have Covid, it is equally the case that the report of Hyams et al may have underestimated them. Many patients presented late in the first wave, sometimes after the expected window of being PCR positive. In addition, in the subsequent waves, we see very similar effects when all the patients have a proven diagnosis, very often having been tested well before they presented to hospital. We are therefore confident that this weakness would not fundamentally later our conclusions, and that to analyse only those patients with positive PCR could introduce bias by preferentially excluding those who presented later with more severe disease. We have added this limitation to the discussion (lines620-623).

5. It would be interesting to analyze if vaccination was a protective factor for those patients admitted to the hospital with an antecedent of immunosuppression, comparing the outcome of vaccinated versus unvaccinated immunosuppressed patients

Authors’ response: This analysis is in fact already published: <https://www.ncbi.nlm.nih.gov/pmc/articles/PMC9111458/#!po=35.7143> Vaccination was protective in the immunocompromised group. We have not repeated the analysis by wave because this reduces the number of patients we can analyse, and it is very difficult to compare across waves because of changes in mortality due to hospital pressures, changes in treatment, and the effect of viral variants.

**Reviewer #2:**

Turtle et al present an analysis from a large national prospective COVID-19 cohort. The large number of patients allows for meaningful sub-group analysis. Only patient data sets linkable to the national immunization management system were analyzed. Two categories were compared: Immunosuppressed, defined by diagnosis (e.g. HIV) and use of immunosuppressive drugs (e.g. steroids pre-admission), with "non"-immunosuppressed patients. The adjusted analysis (with variables commonly associated with worse outcome of COVID-19) shows a remaining elevated risk of death from COVID-19 for immunocompromised patients. This risk, however, is decreasing over time.

The authors conclude that targeted measures such as additional boosters and monoclonal antibodies should be considered. With all its biases, it is one of the largest descriptive studies. A number of remarks/ issues are detailed below.

Concerns/remarks

Even if HIV is counted in the immunocompromised column, it is debatable whether a well-treated HIV patient should be considered immunocompromised. I would be helpful to conduct, if reasonable, some sensitivity analysis with HIV patient alone or without including them into the immunocompromised group.

Authors’ response: We acknowledge this to be a very good point. There are only 498 patients with HIV in this analysis, 2.5% of the total immunocompromised patients in the analysis, so are not likely to change the conclusions. As part of the revision, we have presented ORs for each condition separately (revised Figure 3, previously figure 2 in the earlier version), showing what the effect of each immunocompromising condition is. In this analysis, the number of HIV patients may be too small to be confident of the result, but there is certainly no large effect on the rest of the analyses.

In the conclusion, it would be important to mention "early "therapy with mAB but DAA as well, and prophylaxis, if working with the circulating variant.

Authors’ response: We thank the reviewer for this suggestion. We have modified the end of the discussion accordingly, and have also provided an updated reference and discussion on the use of antibodies as this area continues to evolve (discussion, line 646-647).

**Reviewer #3**

Points of detail
Page 5 The usual definition of adulthood in the UK is ≥ 18. Is this a typo?

Authors’ response: This is in fact not a typo – this is because our paediatric analyses included all patients up to and including the age of 18 years at the time of admission (see: <https://pubmed.ncbi.nlm.nih.gov/35449394/>). Therefore the 18-year-olds have been published already. For this reason, this report includes all the patients aged 19 and over. This difference is a very small number and is not likely to affect our conclusions.

Page 6 I think we might need more explanation of what hospital level deprivation is. Is this a single figure which tries to summarise the entire hospital catchment area? Is it the deprivation index for the middle layer super output area within which the hospital is located? Perhaps more importantly is there any evidence that such a number is related to important features of health outcomes?

Authors’ response: Apologies for this error – we in fact used the lower layer super output area (LLSOA) for determining deprivation based on patient post code. This has been corrected in the text.

Page 7 The frequentist and Bayesian analyses seem to have used a different set of covariates. Vaccination is missing in the Bayesian one. A sentence explaining why might be useful.

Authors’ response: We thank reviewer for picking up this point. We have repeated the Bayesian analysis adjusted by vaccination status, and it shows essentially the same result. The updated plot is now in the manuscript in place of the previous one (figure 4 instead of figure 3, as the patient flow diagram is now moved into the main figures).

Page 7 It would be good to give citations for some of the packages. It is the main way authors get credit. (Disclosure: I am a CRAN package author but have no connection with any of these.)

Authors response: Citations for the Tidyverse, finalfit and brms packages have been added.

Page 19 Typo in caption ‘as both the, incorporating’?

Authors response: Thanks for spotting this – it has been corrected (the word “total” was missing)

Figure 1 The labelling here seems to be in the reverse order to the labelling of the sub–figures. I would have to say I did not find the Euler diagram either easy to read or very enlightening. It is hard to see how much overlap there is assuming that is what the authors intend me to see. The authors might want to consider other packages like eulerr which has some extensive vignettes or, for a different and perhaps more enlightening approach, UpSetR.

Authors response: Thanks for picking up the mislabelling, which has been corrected. We have followed the reviewer’s suggestion and changed the Venn diagram to an Upset plot (and added the package reference). See the updated Figure 2 (previously figure 1)

Figure 2 If the waves differ so much is it informative to present results in sub–figure A collapsed over wave?

Authors response: We felt that this comparison was most important for the primary outcome of death. Although there are marked changes throughout the course of the pandemic in the UK, the changes in both groups over time follow the same trend, though we acknowledge of course that the rate of change for immunocompromised patients is slower, at least for death. We thought that it would be more confusing to present the larger number of sub-categories, especially as we have now been asked by another reviewer to present the categories of immunocompromise separately. We would be happy to present these analyses by wave, but we don’t think it is very informative to present them both by wave and by cause of immune compromise. Because we have already presented the primary outcome by wave, we have elected to leave this as it is, with the addition of a new figure 3A (previously figure 2A) of each outcome measure broken down by cause of immunocompromise.

Supplementary Table S1 Why do so many people in wave 4 appear to be unvaccinated (90+%)? Was vaccination so effective by that time point that it prevented so many admissions?

Authors response: We restricted our analysis to patients that were symptomatic of COVID-19, or at least had symptoms compatible with COVID-19 (the enrolment criteria did not require patients to have symptoms that were definitely attributed to COVID-19). By December of 2021 a very large fraction of the population was either vaccinated (with at least two doses) or had been infected, meaning that many hospital admissions at that point were people admitted for another reason but with COVID-19, rather than because of COVID-19. Omicron was subsequently established to be less virulent than delta. It is plausible at this point that this was indeed the correct fraction of participants admitted with symptoms of COVID.

Supplementary Figure S6 What do the dashed lines represent?

Authors response: The dashed line represents the 95% confidence interval. This has been added to the figure legend.

Points of more substance

Missingness

On page 7 the authors state that since their analysis is predominantly descriptive they are using not taking any steps to deal with missing data. In a sense any true empirical statement can be called descriptive, it describes the world as it is, but that does not mean we can ignore the problem of missing data by default. Clearly I do not find the authors’ justification here very convincing.

The crucial information which we need to evaluate what is happening is the extent to which the analysis sample is reduced. As far as I can see this is not presented anywhere, for instance in Figure S1 (which I would have thought important enough to be in the main article. Since the frequentist and Bayesian analysis appear to have used different predictor variables we need it for both analysis sets. Looking at Table 1 it does seem that some of the missingness may be related to immunocompromise and so there is a potential for bias.

At the least we need to know what variables predict missingness and if it does turn out not to be completely missing at random then imputation would seem indicated. Just to be clear I am not whingeing about the amount of missing. I believe most of the data was collected by non–professional researchers working under difficult circumstances and it is remarkable how little is missing.

Authors’ response: We thank the reviewer for this comment. On reflection we did not address missingness adequately. We have therefore plotted missingness according to several key variables (see figures in the appendix at the end of this document, not included in the manuscript) and conducted a sensitivity analysis, imputing values for those with missing data. This did not substantially affect the results or conclusions of the paper, and is appended as supplementary data (S11 Figure). We have updated the methods (line 265-266) and the results (line 505-511) accordingly.

We have also moved Figure S1 (the patient inclusion/exclusion figure) into the main manuscript (now Figure 1) as per the reviewer’s suggestion. With the inclusion of vaccination in the Bayesian analysis, this sample is now identical so Figure 1 applies all the analysed population.

The Bayesian analysis has been re-run with vaccination as a variable which has not altered the findings. In addition, the Y axis has been labelled. The data are counts, or frequency, which are indicated in the update figure (now figure 4).

Adjusting for comorbidities

I am not clear from Table 1 whether the tally for comorbidity includes the condition(s) which have led to immunocompromise. It is rather suspicious that nobody in that group is free of comorbidity. One of the statements on page 8 seems to confirm that this is indeed a structural zero. Surely if it is genuinely comorbidity it should not include the immune problem itself? Either way I think more thought is needed here. Categorising so that 90+% fall into the 2+ category is too coarse for us to see what the issues are here but adjusting for a variable with very different distributions in the groups reminds me of Lord’s paradox (Lord, 1967).

Authors’ response: We thank the reviewer for this comment. We agree that the categorisation used here was indeed too coarse. We have repeated the comorbidity counting after removal of the immune compromising condition, such that now some patients do have no other comorbidities apart from the condition giving rise to immune compromise. We have repeated the analysis using the revised co-morbidity count.

In addition, we have analysed the data adjusting for the most important individual comorbidities, chronic cardiac, pulmonary and renal disease, in the new odds ratio plots split by reason for immunocompromise. Figure 3 (previously figure 2) has updated according to this revised analysis. This has not changed the conclusions in any way, though, as above, it has refined the degree of risk between the immunocompromising conditions. In Figure 3B, the analysis is adjusted by comorbidity count, as before, but using the number of co-morbidities in addition to the immune compromising condition. We have adjusted the manuscript to make this clearer. (Methods line 275-77, Results line 332-333).

The secondary outcomes

If these outcomes are related to mortality, which there must be evidence that they are, then I find it odd that the odds ratios reported here are less than for mortality itself. Is there some explanation for the differences? I am no expert on critical care so my surprise may be based purely on ignorance.

Authors’ response: the likely reason that the odds ratios are less for the second outcomes is because that although these outcomes (or interventions) are related to mortality, they are also used to prevent mortality. There is likely selection bias in the patients who receive some of these interventions, particularly intensive care and ventilation, towards patients with a greater likelihood of survival. The likely interpretation of this is that use of these interventions has less effect in immunocompromised patients than for immunocompetent patients. However, splitting the groups has revealed that there is substantial heterogeneity in these outcomes according to the cause of immune compromise, and that this difference may be driven by cancer patients who have a higher risk of death, but are less likely to receive some of the interventions. In addition to being related to mortality, these interventions can also be withheld in some instances due to the perception of clinical futility. Therefore, the patients who are not invasively ventilated, for example, comprise those who are well enough that they do not need it, alongside those who are so ill that it is deemed to be futile.

**Comments from the academic editor:**
Indeed, the paper could contribute important information. 
I would agree with inviting the author to submit a major revision. I would like them to respond to the following:
1.      From a clinical point of view, it is not helpful to lump all causes of immunocompromise together. There are >20,000 immunocompromised patients in the study. Surely it would be possible to examine whether the outcomes are better or worse, according to the reason for immunocompromise. As Reviewer #2 points out, people living with HIV in the UK are usually well-controlled on antiretroviral therapy. Maybe those admitted are not. Anyhow, I would like to know how mortality differed according to the reason for immunocompromise.

Authors’ response: thanks for this suggestion – we have done this and presented the new analysis in Figure 3A (previously figure 2A). This has shown significant heterogeneity between the groups, with cancer patients in particular having poor outcomes. We have modified the results “outcomes” section (lines 390-475) and the discussion (lines 627-633) accordingly. We have also moved what was previously Table S4 to become table 2 – this shows the raw numbers of events of each outcome by cause of immune compromise. The previous table 2 is now S9 Table.

2.      Fig S1, The authors excluded 51,903 patients reported to be ‘asymptomatic’ – is this asymptomatic on admission or throughout the course of infection? Did no patients asymptomatic on admission subsequently develop symptoms and die?

Authors’ response: For most of the patients in the study, the CRF only collected data on symptoms on admission, and not on other days. Patients who were admitted to hospital and had a positive SARS-CoV-2 PCR but did not have symptoms of COVID-19 were admitted to hospital for another reason. This other condition (which we do not know) would therefore have driven their outcome, so they were excluded from the analysis. We did not collect information on the development of symptoms of COVID-19 whilst in hospital. However, even if we had, we would not have known if they passed the threshold for admission independently of the other condition.

3.      Vaccination, lines 193-9 – there is a detailed explanation of how vaccination status is coded – I can’t see how this is incorporated into any analysis

Authors’ response: This was incorporated into the analysis as a multi-level variable in the logistic regression. A sentence in the methods has been added to clarify this (line 238-239).

4.      Missing data, line 217 – I fully agree with Reviewer #3, we need to know more about the missing data– are thy the same patients? What would be the likely direction of under-or over-estimation?

Authors’ response: See response to reviewer #3. We have performed a sensitivity analysis to account for missing data, and have provided visualisation of missingness in an appendix here. This has not changed the conclusions of the manuscript.

5.      Statistical analysis, line 238 – can the authors explain why they do a Bayesian analysis for the changes over time? Can they also explain Figure 3 better? What is y (left had vertical axis)? X axis is labelled ‘Absolute risk difference for death’ – what are the units for this? The figure only has proportions, but the text reports %.

Authors’ response: We performed this analysis to allow probabilistic interpretations of absolute risk differences. We have added a line to this effect in the methods (line 283). The figure has been updated with the units and a Y axis label. The new figure is now figure 4.

6.      Discussion (main text and abstract conclusions): I don’t think the conclusions are really supported by the results – the conclusions are that more vaccine doses and monoclonal antibody treatments are needed – but there is no analysis showing that these improve outcomes in immunocompromised people., only that mAbs are not used very much and most people admitted are unvaccinated (with no difference between immunocompetent and immuocompromised).

Authors’ response: Thanks for this comment – we agree that in the earlier version we overstated these conclusions. We have toned this down, added the comment about direct acting antivirals as suggested by reviewer #2 (discussion lines 643-647). We have also updated the reference for monoclonals to a more recent report (line 644, ref 34), showing that although some monoclonal preparations have lost neutralising activity against the omicron variant, this is not a one directional process and some omicron variants have emerged which have re-gained susceptibility to existing treatments. In light of the lack of difference between numbers unvaccinated we have adjusted this sentence to ensuring vaccine uptake (line 647).

**PLOS Medicine style changes:**

PLOS Medicine requires that the de-identified data underlying the specific results in a published article be made available, without restrictions on access, in a public repository or as Supporting Information at the time of article publication, provided it is legal and ethical to do so. Please see the policy at http://journals.plos.org/plosmedicine/s/data-availability and FAQs at http://journals.plos.org/plosmedicine/s/data-availability#loc-faqs-for-data-policy

For each data source used in your study:
a) If the data are freely or publicly available, note this and state the location of the data: within the paper, in Supporting Information files, or in a public repository (include the DOI or accession number).
b) If the data are owned by a third party but freely available upon request, please note this and state the owner of the data set and contact information for data requests (web or email address). Note that a study author cannot be the contact person for the data.
c) If the data are not freely available, please describe briefly the ethical, legal, or contractual restriction that prevents you from sharing it. Please also include an appropriate contact (web or email address) for inquiries (again, this cannot be a study author).

Authors response: The data used for this analysis are not freely available because the ISARIC CCP-UK dataset, and the NHS Digital data used to determine vaccination status, contain personally identifiable data. Arrangements for access to the ISARIC data are handled by the ISARIC4C Independent Data and Material Access Committee, details at <https://isaric4c.net/sample_access/>. NHS Digital data can be access by application to the NHS Digital Data Access Request Service (DARS) at <https://digital.nhs.uk/services/data-access-request-service-dars>. This has been added to the manuscript.

Did your study have a prospective protocol or analysis plan? Please state this (either way) early in the Methods section.
a) If a prospective analysis plan (from your funding proposal, IRB or other ethics committee submission, study protocol, or other planning document written before analyzing the data) was used in designing the study, please include the relevant prospectively written document with your revised manuscript as a Supporting Information file to be published alongside your study, and cite it in the Methods section. A legend for this file should be included at the end of your manuscript.

Authors response: ISARIC CCP-UK has a prospective protocol, which can be accessed online at: <https://isaric4c.net/protocols/>. This is referenced in the manuscript.

b) If no such document exists, please make sure that the Methods section transparently describes when analyses were planned, and when/why any data-driven changes to analyses took place.
c) In either case, changes in the analysis-- including those made in response to peer review comments-- should be identified as such in the Methods section of the paper, with rationale.

See above

ABSTRACT
Please structure your abstract using the PLOS Medicine headings (Background, Methods and Findings, Conclusions). Please combine the Methods and Findings sections into one section, “Methods and findings”.

Abstract Methods and Findings:
Please ensure that all numbers presented in the abstract are present and identical to numbers presented in the main manuscript text.
Please include the study design i.e. prospective, observational), length of follow up, and main outcome measures.
Please include the important dependent variables that are adjusted for in the analyses.
In the last sentence of the Abstract Methods and Findings section, please describe the main limitation(s) of the study's methodology.

Authors response: The abstract has been structured as requested. The numbers have been checked for consistency. In addition the key denominators have been repeated in the text where relevant to enhance clarity. This was a prospective cohort study – this is in the title but has been added to the abstract and the methods. Important dependent variables are given in the abstract.

Abstract Conclusions:
Line 80: “...additional vaccine doses and monoclonal antibodies” any role for non-pharma preventative interventions (masks, distancing in-hospitals etc)? might be worth a mention

Authors response: The suggested phrase has been added to the abstract.

At this stage, we ask that you include a short, non-technical Author Summary of your research to make findings accessible to a wide audience that includes both scientists and non-scientists. The Author Summary should immediately follow the Abstract in your revised manuscript. This text is subject to editorial change and should be distinct from the scientific abstract. Please see our author guidelines for more information: https://journals.plos.org/plosmedicine/s/revising-your-manuscript#loc-author-summary

Authors response: The authors’ summary has been added after the abstract.

METHODS and RESULTS
Please define "lost to follow-up" as used in this study. Other reasons for exclusion should be defined.
Please define the length of follow up (eg, in mean, SD, and range).

Authors response: This was a hospital based study of acute illness, there was therefore no period of follow up as such. Hospital data teams were asked to complete the CRF on death, discharge or transfer to another facility, even if this occurred after 28 days. However, for this analysis, only the 28 day outcome was used. Patients were considered to be alive if the 28 day outcome was missing. Reasons for exclusion are detailed on page 6 (lines 189-195), these were: absence of a linkable NHS number, re-admission, and erroneous admission dates. The text has been amended at line 195-197.

Line 270: “...(median age 72.0. IQR (62.0 to 79.7))” please amend the presentation of these data to the following: (median age 72.0 [IQR 62.0-79.7]) and throughout the manuscript
Similarly line 285: (n=6,607 (30%)) (n=6,607 [30%]) and throughout

Authors response: This has been changed throughout for any brackets nested within another set of backets. The deleted round brackets have not been tracked (accepted) for reasons of clarity.

Line 301: Please provide the actual numbers of events for the outcomes, not just summary statistics , %, or ORs. Please check and amend throughout

Authors’ response: This has been checked and changed throughout where necessary.

Line 341: please present numerators and denominators for percentages – please check and amend throughout

Authors’ response: In the severity of illness section, (now line 378-381), for the sake of brevity, the numbers and percentages have been omitted. In this section giving all the data would make the section substantially longer without improving the clarity. We would of course be happy to put the numbers in here if the editor or reviewers prefer, this adds three additional sentences.

For the following analyses: outcomes of hospitalised immunocompromised patients, compared with immunocompetent patients presented in figure 2 please also provide unadjusted analyses

Authors’ response: Thanks for giving us the opportunity to sign post this better. The univariate analysis is presented in S10 Figure for the primary outcome, and. We have re-worded this more clearly at line 487.

FIGURES
Please provide titles and legends for all figures (including those in Supporting Information files).

Authors’ response: These have been added.

Figure 2 – as above, please also present unadjusted analyses (you may wish to consider the use of a table)

Authors’ response: This has been done as above, in S10 Figure.

TABLES
Table S4: Please define numbers and those in brackets

Authors’ response: These are numbers (%) - this has been added, the table has been moved to become table 2 to account for the presentation of the analysis by cause of immune compromise.

SUPPLEMENTARY INFORMATION

Please provide titles AND legends for each individual table and figure in the Supporting Information.
You have included a CONSORT diagram (figure S1) which would be more relevant for an RCT. The information contained within is helpful to the reader and I have no objection to it remaining but would suggest a revised title and an appropriately associated legend

Authors’ response: The title has been updated and a legend added. Note also, in response to reviewer #3, that this has been moved into the main figures. It is also the same sample as is used in the Bayesian analyses.

Please ensure that the study is reported according to the STROBE guideline and include the completed STROBE checklist as Supporting Information. Please add the following statement, or similar, to the Methods: "This study is reported as per the Supporting Information. Please add the following statement, or similar, to the Methods: "This study is reported as per the Strengthening the Reporting of Observational Studies in Epidemiology (STROBE) guideline (S1 Checklist)." The STROBE guideline can be found here: http://www.equator-network.org/reporting-guidelines/strobe/ When completing the checklist, please use section and paragraph numbers, rather than page numbers.

Authors’ response: The STROBE checklist has been uploaded with the revision. The STROBE statement is in the methods, and has been updated to refer to the checklist in the supplementary information.

REFERENCES
Please select the PLOS Medicine reference style in your citation manager. In-text reference call outs should be presented as follows noting the absence of spaces within the square brackets, “...symptomatic [2,8].” Please ensure no more than 6 author names are listed in the bibliography before et al (if there are more than 6 contributing authors)

Authors’ response: The reference format has been changed.

Appendix – missing data

Missing data by age

Missing data by wave

Missing data by deprivation

Missing data by immunocompromise

Missing data by 4C mortality score

Missing data by sex
