# Supplementary material for: Outcome of COVID-19 in hospitalised immunocompromised patients: An analysis of the WHO ISARIC CCP-UK prospective cohort study
Source: PLoS Med. 2023 Jan 31;20(1):e1004086. doi: 10.1371/journal.pmed.1004086 (PMC9928075; doi:10.1371/journal.pmed.1004086)
Supplement: Attachment — Submitted filename: Response to review v2.docx [file pmed.1004086.s015.docx]

COMMENTS FROM THE ACADEMIC EDITOR
I would particularly like to see the authors include some of their explanations to the reviewers as text in the article itself. They might have done some of this, but since they omit line numbers in many of their comments, I can't tell.

We apologise for not putting in line numbers of all the changes in the response to reviewers. We have signposted here to the significant changes, and have put in line numbers as well as indicating the altered text.

1. Response to Reviewer #1, comment 2 (adjustment for pharmacological treatment) please sign post to where this is addressed

Apologies for not signposting this clearly enough - this is addressed at line 495 – 497 in the clean version of R1, lines 540 – 542 in the tracked version of R2 (no change to text in R2).

*“Missing data in the medical and drug histories means that we may have underestimated the overall effect size by categorising some immunocompromised patients as immunocompetent. This also prevented us from examining any relationship between the treatments given for COVID-19 and immunocompromise.”*

Underlined text is the new addition.

2. They say they mean to start from age 19 because they already published the paediatric data. Please state this, under 'Participants' and cite the publication (see comments below also)

This has been added to the methods section, lines 190 – 191 of tracked R2:

*“Participants 18 years and under were excluded because most of these cases had been previously reported [REF 18 Swann et al].”*

See also below, METHODS and RESULTS.

3. Reviewer #2, 'Upset plot' - can they delete 'Upset'? I don’t know what that is and I don't think it's helpful

Thanks for picking this up – we agree the term is not very informative and have removed it from the legend for figure 2. (We have left the name UpSetR in the methods as this is the name of the R package. I have no idea why it’s called “Upset” either.)

4. Reviewer #2, S1 Table – regarding the proportion of unvaccinated people – please add an appropriate explanation to the discussion.

We have added the following more detailed explanation to the discussion of the proportion of unvaccinated people in the study. The following new text has been added (lines 517 – 524 of tracked R2):

*“This difference was particularly high by the fourth wave, when most of the symptomatic patients were unvaccinated in both groups, likely reflecting the reduction in severity of the omicron variant [28]. We have previously showed that immunocompromised patients are enriched in patients admitted after vaccination [29], although vaccination is of benefit in this patient group [30]. We cannot directly compare vaccine efficacy as we lack the denominator population, and we cannot estimate how many patients avoided admission because of vaccination.”*

Two new references have been also added to support statements in this section:

Ref 28 – Nyberg et al. Comparative analysis of the risks of hospitalisation and death associated with SARS-CoV-2 omicron (B.1.1.529) and delta (B.1.617.2) variants in England: a cohort study. The Lancet. 2022;399(10332):1303-12

Ref 30 – Britton et al. Effectiveness of COVID-19 mRNA Vaccines Against COVID-19-Associated Hospitalizations Among Immunocompromised Adults During SARS-CoV-2 Omicron Predominance - VISION Network, 10 States, December 2021-August 2022. MMWR Morb Mortal Wkly Rep. 2022;71(42):1335-42

They have dealt with all my own comments.

** In addressing point 4 above, the editorial team suggest that you take care with your wording as the explanation in your rebuttal (without the question) could be confused with justification simply for the number of number of admissions i.e. please be sure to refer to those ppts as unvaccinated

Please see the newly added text above. We have re-written this section rather than pasting in the text from the rebuttal – we hope this makes it sufficiently clear what we are able to conclude, and not conclude, about vaccine efficacy from this population.

ABSTRACT
In the last sentence of the methods and findings section please include the main limitations of your methodology.

We have added the following sentence at the end of the methods and findings section (lines 83 – 85, tracked R2):

*“The study is limited by a lack of detailed drug data prior to admission, including steroid doses, meaning that we may have incorrectly categorised some immunocompromised patients as immunocompetent.”*

Please remove the funding statement from the end of the abstract and include only in the submission form.

This has been removed.

AUTHOR SUMMARY
Thank you for including an author summary. Please structure as follows, separating each paragraph into (1-4) individually bulleted points below each sub-heading:

Why Was This Study Done? 
Authors should reflect on what was known about the topic before the research was published and why the research was needed.

What Did the Researchers Do and Find? 
Authors should briefly describe the study design that was used and the study’s major findings. Do include the headline numbers from the study, such as the sample size and key findings.    

What Do These Findings Mean? 
Authors should reflect on the new knowledge generated by the research and the implications for practice, research, policy, or public health. Authors should also consider how the interpretation of the study’s findings may be affected by the study limitations.

We have split up the author summary according to this scheme. We have also added to the bottom the following text (lines 127 – 130, tracked R2):

*“Clinicians and policy makers should be aware of the increased risk of death in this patient group. Targeted interventions such as antiviral treatments, antibodies, and non-pharmaceutical interventions should continue to be used in this patient group.”*

INTRODUCTION 
Line 146: please remove the word “differed” it is redundant here

Thanks for spotting this typo – this word has now been removed.

METHODS and RESULTS
Line 374 (for example): suggest the following - “(OR 2.0, 95% CI [1.87,2.25], p<0.001)” note the addition of parentheses around CIs and suggest considering the use of a comma instead of a hyphen (hyphens can become confused with reporting of negative values).  Please check and amend throughout, including in the abstract

Thanks – this change has been made throughout.

Reviewer #3 response to age inclusion (18 Vs 19 year olds) “paediatric analyses included all patients up to and including the age of 18 years”– thank you for clarifying this point.  It would be helpful to add this clarification to the appropriate part of the manuscript we suggest including this under the sub-heading “participants”

We have added the following line to the methods section (lines 190 – 191, tracked R2):

*“Participants 18 years and under were excluded because these cases had been previously reported [18].”*

Ref 18 has been added – Swann et al. Comparison of UK paediatric SARS-CoV-2 admissions across the first and second pandemic waves. Pediatr Res. 2022. Epub 2022/04/23

DISCUSSION
Please present and organize the Discussion as follows: a short, clear summary of the article's findings; what the study adds to existing research and where and why the results may differ from previous research; strengths and limitations of the study; implications and next steps for research, clinical practice, and/or public policy; one-paragraph conclusion.

We feel that the first paragraph does give a succinct summary of the findings. We have made one small modification (line 451, tracked R2):

*“Over time, mortality….”*

New text is underlined.

The component parts all seem to be there, but a little re-structuring and a clearly defined conclusion would be of benefit

The discussion has been modified according to the suggestion. The details can be seen in the tracked changes. We have added a final paragraph with a clearer conclusion (lines 601 – 606, tracked R2):

*“Despite the benefits of vaccination, immunocompromised patients still lag behind the general patient population in the improvements in outcomes after hospitalisation. Clinicians and policy makers should be aware of the increased risk of death in this patient group. Targeted interventions such as antiviral treatments, antibodies, and non-pharmaceutical interventions should continue to be used for immunocompromised patients with COVID-19.”*

Please remove the data availability statement from the end of the discussion (line 554) and include only in the submission form, it will be compiled as meta-data.

This has been removed.

REFERENCES
Line 131 (for example): “[13, 14].” Please remove spaces between citations as follows: “[13,14]”.
Please add reference to the paediatric data already published which includes 18 year olds (see also academic editor comment above)

These have been done.

SOCIAL MEDIA
To help us extend the reach of your research, please provide any Twitter handle(s) that would be appropriate to tag, including your own, your co-authors’, your institution, funder, or lab. Please respond to this email with any handles you wish to be included when we tweet this paper.  Please include these in the submission form when you re-submit your manuscript

Please see below:

@abdocherty79

@Tom_Drake1

@Thushan_deSilva

@cancermedic

@stephen_aston

@AlexRichter3

@DrToniHo

@evusheld4theuk

@clarkdrussell

@CCPUKstudy

@ProfCalumSemple

@kennethbaillie

@HPRUezi

@LivUni

These will also be sent by email.


Comments from Reviewers:

Reviewer #1: The authors have appropirately addressed the points raised in my previous report. This is an important article confirming immunosupressed as a group at risk of poor outcomes following infection by SARS-CoV-2. These results support those interventions aimed at preventing uncontrolled viral replciation in these patients (vacccines, early antiviral treatment, (active) monoclonal antibodies)


Reviewer #2: The authors have carefully responded to all concerns raised, and improved the manuscript.


Reviewer #3: The authors have addressed all my points.

Michael Dewey
